# Hypofractionated helical intensity-modulated radiotherapy of the prostate bed after prostatectomy with or without the pelvic lymph nodes - the PRIAMOS trial

**DOI:** 10.1186/1471-2407-12-504

**Published:** 2012-10-31

**Authors:** Sonja Krause, Florian Sterzing, Dirk Neuhof, Lutz Edler, Juergen Debus, Klaus Herfarth

**Affiliations:** 1 Department of Radiation Oncology, University Hospital Heidelberg, Im Neuenheimer Feld 400, 69120, Heidelberg, Germany; 2 Department of Biostatistics, German Cancer Research Center, Im Neuenheimer Feld 280, 69120, Heidelberg, Germany

**Keywords:** Prostate cancer, Radiotherapy, Hypofractionation, Helical tomotherapy, Prostate bed, Pelvic lymph nodes

## Abstract

**Background:**

While evidence on safety and efficacy of primary hypofractionated radiotherapy in prostate cancer is accumulating, data on postoperative hypofractionated treatment of the prostate bed and of the pelvic lymph nodes is still scarce. This phase II trial was initiated to investigate safety and feasibility of hypofractionated treatment of the prostate bed alone or with the pelvic lymph nodes.

**Methods/design:**

A total of 80 prostate cancer patients with the indication for adjuvant radiotherapy will be enrolled, where 40 patients with a low risk of lymph node involvement (arm 1) and another 40 patients with a high risk of lymph node involvement (arm 2) will each receive 54 Gy in 18 fractions to the prostate bed. Arm 2 will be given 45 Gy to the pelvic lymph nodes additionally. Helical Tomotherapy and daily image guidance will be used.

**Discussion:**

This trial was initiated to substantiate data on hypofractionated treatment of the prostate bed and generate first data on adjuvant hypofractionated radiotherapy of the pelvic lymph nodes.

**Trial registration:**

ClinicalTrials.gov; NCT01620710

## Background

With 58.000 newly diagnosed cases every year in Germany, prostate cancer is the most common cancer of men in Germany. As the proportion of men older than 60 years is estimated to rise with the demographic shift up to 37% in 2050, prostate cancer will gain more and more epidemiological and economical significance [1]. For patients with localised prostate cancer, radical prostatectomy and definitive radiotherapy are primary treatment options. The guidelines of the German Society of Urology strongly recommend postoperative radiotherapy for patients with stage pT3 and positive margins. In addition, adjuvant radiotherapy should be considered for stage pT3 with negative margins and pT2 with positive margins [1]. In the case of a recurring PSA elevation, salvage radiotherapy should be started early [2].

Three phase III trials demonstrated a superior biochemical recurrence-free survival (PSA-BFS) for adjuvant radiotherapy compared to surgery alone: EORTC 2291 [3], SWOG 8794 [4] and ARO 96–02 [5]. Additionally, an update of SWOG 8794 trial exhibited an advantage in overall survival for adjuvant radiotherapy [6]. While the toxicity profile of definitive radiotherapy is well characterised, data on side effects of adjuvant irradiation is relatively sparse. Patients in the EORTC trial showed more side effects in the radiotherapy arm, however, the rate of NCI CTC AE toxicities grade 3 or higher was comparable to the control arm. In the ARO trial, 3% of the irradiated patients suffered from acute, and 2% of late grade 3 bladder toxicities and 10% showed grade 2 late rectal toxicity.

Irradiation of the pelvic lymph nodes (whole pelvis radiotherapy, WPRT) in patients with a high risk of lymph node involvement according to the Roach formula is still subject to discussion. While some randomised trials could demonstrate a benefit for the WPRT in a definitive setting (RTOG 94–13 [7]), others could not (RTOG 77–06 [8], GETUG-01 [9]). For postoperative WPRT, Spiotto et al. [10] showed in a retrospective analysis of 160 high-risk patients an improved 5-year BFS.

In recent years, hypofractionated radiotherapy, i.e. the treatment with an increased daily dose, has gained importance in prostate cancer radiotherapy. The radiation-induced death of mammalian cells is a function of total dose, daily dose and treatment duration. The radiation sensitivity of normal tissues and cancer cells is described by the α/β value using the linear-quadratic model. The α/β value appears to be high (≥ 10 Gy) for so-called early-reacting tissues (e.g. skin, mucosa and most tumor cells) and low (< 5 Gy) for late-reacting tisuses (e.g. spinal cord and bone). Differences in the α/β values between normal tissues and tumor cells are the basis for developing fractionation regimes in radiation oncology. For prostate cancer cells, in vitro studies and retrospective analyses have indicated an α/β value as low as 1.5 Gy (and thus lower than the respective values for rectum and bladder), implying that patients with prostate cancer might benefit from treatment with high daily doses [11]. For primary radiotherapy, several non-randomised prospective trials have demonstrated comparable rates of acute and late toxicity and, in particular, similar efficacy when comparing hypofractionated and conventionally fractionated radiotherapy: Kupelian et al. [12] saw in a phase I/II trial when treating with 70 Gy in 2.5 Gy single doses a low rate of rectal toxicity and a biochemical disease control comparable to patients treated with conventional fractionation. An early analysis of an Italian phase III trial [13] comparing 62 Gy in 3.1 Gy single doses to 80 Gy in conventional fractionation reported a 3-year grade 2 rectal toxicity of 17% vs. 16% and grade 3 genitourinary (GU) toxicity of 14% vs. 11%. 3-year-BFS was significantly higher in the hypofractionated arm (87% vs. 79%, p=0,035). In line with these data, investigators of a randomised phase III trial [14] comparing 55 Gy in 2.75 Gy single doses with 64 Gy in 2 Gy single doses saw no difference in late gastrointestinal (GI) and GU toxicity, while the BFS rate at 90 months was statistically significantly higher in the hypofractionated group (53% vs. 34%).

While hypofractionated regimen are beginning to be integrated into clinical routine in first line treatment, data on postoperative hypofractionated radiotherapy is still sparse. Kruser et al. observed low toxicity rates in 108 patients treated with 65 Gy in 2.5 Gy single dose: Only one patients suffered from acute grade 3 GU toxicity and no acute grade 3 GI toxicities occurred. No patient suffered from any late grade 3 toxicity [15].

While the early trials on prostate radiotherapy have been conducted with conventional radiation techniques, it has been demonstrated that the dose to the prostate can be escalated with intensity-modulated radiotherapy (IMRT) without the risk of higher toxicity [16,17]. In addition, sophisticated imaging devices have been implemented in daily routine that can ensure exact dose application. Helical Tomotherapy (HT) constitutes a fusion of a linear accelerator with a spiral CT scanner, offering the possibility of MV-CT imaging with good soft tissue contrast before each treatment fraction combined with IMRT treatment with sharp dose gradients [18,19].

We initiated this phase II trial to investigate the toxicity profile and efficacy of a hypofractionated postoperative radiotherapy of the prostate bed with or without the inclusion of the pelvic lymph nodes using HT under daily image guidance.

## Methods/design

### Study design

**Figure 1:**
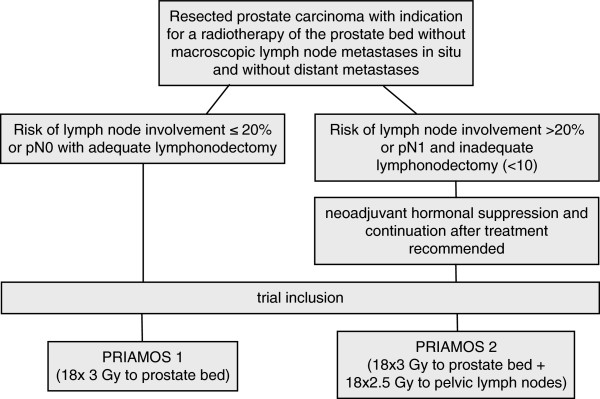
Trial flowchart.

The PRIAMOS trial is a single-center, non-randomised prospective two-arm prospective phase II trial. Patients will be stratified according to their risk of lymphatic involvement into either hypofractionated radiotherapy of the prostate bed alone with 54 Gy in 18 fractions of 3.0 Gy each (low risk of lymph node involvement, arm 1, PRIAMOS 1) or additional simultaneous treatment of the pelvic lymph nodes with 45 Gy in 18 fractions of 2.5 Gy (high risk of lymph node involvement, arm 2, PRIAMOS 2). Patients eligible for the PRIAMOS trial have to present with the indication for a radiotherapy of the prostate bed. Patients with a risk of lymph node involvement of >20% according to the Roach formula [20] (2/3 PSA + [Gleason - 6] x 10) with inadequate lymphadenectomy (< 10 lymph nodes) and patients with positive resected nodes (pN1) are attributed to arm 2 and receive a simultaneous treatment of the pelvic lymph nodes. For the trial flowchart, see Figure 1.

### Study objectives

The main objective is to demonstrate the safety and feasibilty of a hypofractionated helical IMRT of the prostate bed with (PRIAMOS 2) or without (PRIAMOS 1) the pelvic lymph nodes. Treatment safety will be judged by the incidence of NCI CTC AE grade 3–4 toxicity and by occurrence of treatment discontinuation.

Secondary objectives are BFS, quality of life (QoL) and clinical symptoms. A biochemical recurrence is defined as 3 consecutive rises in PSA levels (ASTRO criteria). QoL is investigated using the EORTC QLQ-C30 and QLQ-PR25 questionnaires; symptoms and toxicities will be graded using the NCI CTC AE version 4.0 grading criteria.

### Trial organisation

The PRIAMOS trial is a single-center, investigator-initiated trial carried out by the Department of Radiation Oncology, University Hospital of Heidelberg, Germany.

### Patient selection: inclusion criteria

Patients meeting all of the following criteria are eligible for the PRIAMOS trial:

resected prostate carcinoma with histological grading (Gleason Score)

status post prostatectomy for a pT3 carcinoma and/or R1/2 resection or PSA recurrence after prostatectomy (2 consecutive PSA rises)

PSA recurrence ≥ 1 ng/ml: CT/PET/MRI imaging excluding pathological lymph nodes

Karnofksy performance score ≥ 70%

age 18–80 years

only arm 2: antihormonal therapy for 2 months prior to radiotherapy and continuation of hormonal suppression after radiotherapy recommended

written informed consent

### Patient selection: exclusion criteria

Patients meeting at least one of the following criteria are not eligible for the PRIAMOS trial:

patient‘s refusal

patient‘s inabillity to give informed consent

stage IV (distant metastases)

lymph node involvement outside the pelvis

severe wound complications after laparatomy

only arm 2: severe lymphoedema of the legs, elephantiasis, postthrombotic syndrome

decompensated pulmonary, cardiovascular, metabolic, hematopoetic, coagulatory or renal comorbidities

known other malignant disease with distant metastases

prior pelvic irradiation

participation in another clinical trial that might compromise the results of the PRIAMOS trial or the other trial

### Statistical design

Each of the two arms (PRIAMOS 1 and PRIAMOS 2) is designed an independent single stage phase II trial with the safety and feasibility the respective treatment as primary endpoint, precisely defined as the proportion SDR of patients with no NCI CTC AE grade 3–4 toxicity and no discontinuation of treatment during the full set of 18 fractions by any reasons in the intent-to-treat (ITT) population. The ITT population is defined by compliance with the in- and exclusion criteria and not dropping out within one week after start of treatment.

The study hypothesis is chosen as H1: SDR≥ 95% to be tested versus the null hypothesis of H0: SDR≤ 80% using a single arm exact binomial test for proportions. In order to test H0 versus H1 one-sided at the significance level of 0.10 (10%) n= 32 evaluable patients are required to achieve a power of 90%, when using PASS software and A’Hern [21] and choosing the type I and the type II error identical for such a phase II trial. In order to compensate possible drop out between informed consent and treatment for at least one week n=40 patients are planned to be recruited per arm.

No formal interim analysis is scheduled. However, recruitment is put on hold in an arm when the proportion SDR surpasses the critical number of 4. The study coordination team will then decide on stop of recruitment of the respective as well as the other trial arm.

Statistical evaluation of the primary endpoint as well as of the secondary endpoints BFS, QoL, overall survival and detailed safety analysis will be performed separately for each arm, for the ITT (SDR, BFS, QoL) as well as for the per protocol (PP; SDR,BFS) and the safety (SF; QoL, NCI CTC AE) population. Statistical comparisons between the two trial arms are performed exclusively with descriptive intention.

### Investigation schedule

#### Therapy indication

For each patient, the decision to treat the prostate bed is made by a radiation oncologist based on the S3-guideline of the German Society of Urology [1]. Indications for a postoperative or salvage radiotherapy of the prostate bed are a stage pT3, microscopically or macroscopically positive resection margins (R1/2) or a PSA recurrence after prostatectomy. For patients with a risk of lymph node involvement according to the Roach formula (2/3 PSA + [Gleason - 6] x 10) > 20% and inadequate (<10) lymphadenectomy, the additional simultaneous treatment of the pelvic lymph nodes is indicated.

#### Pre-Therapeutic examinations

Prior to treatment, a complete medical history including the surgical report and the histological examination is taken. In the case of a PSA ≥ 1 ng/ml, a recent CT, MRI or PET ruling out pathological lymph nodes, and in the case of a PSA ≥ 3 ng/ml a bone scintigraphy ruling out bone metastases have to be performed before the start of an antihormonal treatment. A baseline QoL assessment as well baseline symptoms are recored prior to start of treatment. At the first treatment day, PSA levels and haemogram are measured.

#### Planning of radiotherapy

Before start of radiotherapy, a planning CT in 3 mm slices is taken without contrast medium. The patient is positioned using a knee and foot support. During planning CT scan and when treatment fractions administered, the bladder should be full and the rectum empty.

The PTV-P (planning target volume-prostate bed) comprises the prostate bed including the bottom of the bladder and the anterior rectal wall with a security margin of 0.5 cm. For arm 2, an additional PTV-L (planning target volume-lymph nodes) including the obturatory, perirectal, internal and external iliac, common iliac and presacral lymph nodes according to the RTOG guidelines [22] with a 0.5 cm margin is contoured. Intersections of PTV-P with the rectum and of PTV-L with the small bowel are constructed to avoid hotspots in rectum and small bowels. Rectum, bladder, small bowel and femoral heads are contoured as organs at risk. A dose of 54 Gy in 3 Gy daily fractions is prescribed to 95% of the PTV-P. Additionally, in arm 2, a dose of 45 Gy in 2.5 Gy fractions is prescribed to 95% of the PTV-L. Assuming an α/β of 1.5 Gy for prostate cancer, this translates to an equivalent dose of 69.4 Gy to the prostate bed and 51.4 Gy to the lymph nodes. As planning constraints, a maximum dose to the small bowels of 45 Gy, even if resulting in a reduced coverage of PTV-L, has to be respected. Dose to all organs at risk should be kept as low as possible, and the TD 5/5 of the respective organs must not be exceeded.

#### Radiation therapy

Radiotherapy is performed as a helical IMRT using a Tomotherapy® Hi-Art or HD unit (Accuray Inc., Sunnyvale, CA, USA). Before each treatment, a 3.5 MV fan beam CT is performed and matched to the planning CT. If needed, the patient positioning is corrected. If necessary, rectal and bladder filling are adjusted before the start of treatment. Treatment is performed in 18 daily fractions (Monday-Friday only). Radiotherapy is conducted on an outpatient basis with the possibilty of hospitalisation if required.

#### Monitoring during treatment

During treatment, constant clincal monitoring and, if necessary, supportive therapy, is guaranteed. Once weekly, symptoms and toxicities are assessed and the haemogram is measured. PSA is not measured during treatment. The following symptoms and toxicities are of special clinical interest: urinary incontinence, nocturia, cystitis, stool frequency, stool incontinence, diarrhoea, proctitis, lymphoedema and virility.

#### Follow-up

A first PSA measure after treatment is taken 6 weeks after the end of radiotherapy and then every three months. Toxicity and symptoms are recorded at 6 weeks, 6 months, 12 months, 18 months and 24 months after radiotherapy. QoL is assessed at 6 weeks, 6 months and 24 months after radiotherapy using the EORTC questionnaires.

#### Duration of the study

Patient accrual started in March 2012; the end of accrual is planned for March 2014. Analysis of the primary endpoint for each separate arm is planned for 24 months after the first treatment day of the last patient, and the end of the study is defined as the end of the last patient‘s observation period.

#### Data handling, storage and archiving of data

All clinical and laboratory data and radiotherapy plans will be documented by the investigators or an authorised member of the study team in the patient‘s medical record and in the case report forms (CRF). The data will be stored and archived according to §13 of the German GCP Regulation and §28c of the German X-Ray Regulation (StrSchV) for at least 30 years after the trial termination.

#### Ethics, informed consent and safety

The final trial protocol was approved by the ethics committee of the Medical Faculty of the University of Heidelberg, Germany (Nr.: S-599/2011). An expert committee of the German Society of Radiation Oncology (DEGRO) approved the protocol and stated that the trial did not need a vote of the Federal Office for Radiation Protection (Bundesamt für Strahlenschutz, BfS). The study complies with the Helsinki Declaration in its recent German version. The trial is also carried out in keeping with local legal and regulatory requirements.

## Discussion

In definitive radiotherapy for prostate cancer, hypofractionation is being integrated into standard treatment regimen in an increasing amount of treatment centers. Similar toxicity rates and at least equal efficacy have been affirmed by several non-randomised and randomised trials [12,14,23]. Moreover, several large randomised phase III trials are recruiting currently or have just finished recruiting: A trial of the Fox Case Cancer Center compares 76 Gy in 2 Gy fractions vs. 70.2 Gy in 2.7 Gy fractions, while a NCIC trial randomises patients to 78 Gy in 2 Gy fractions or 60 Gy in 3 Gy fractions. RTOG 0415 randomises low-risk patients to 73.8 Gy in 1.8 Gy fractions or 70 Gy in 2.5 Gy fractions. Should the α/β for prostate cancer be 10 Gy, both arms would be isoeffective. With an α/β of 1.5 Gy, however, the biologically effective dose (BED) would be 15% higher in the hypofractionated arm and should result in a better clinical outcome.

While hypofractionated radiotherapy in the definitive setting has been investigated extensively, data on postoperative hypofractionated treatment is sparse. An Italian phase I-II trial [24] treated 50 patients with helical IMRT using 58 Gy in 2.8 Gy fractions with excellent safety outcomes that were comparable to their data on conventionally fractionated treatment. Twelve percent of their patients suffered from acute grade 2–3 GU side effects and 4% from acute grade 2 intestinal side effects. No acute grade 2 proctitis and no late grade 2 GI sequelae were reported. Similarly low toxicity rates have been demonstrated by Kruser and colleagues by treating the prostate bed with 65 Gy in 2.5 Gy fractions [15]. To our knowledge, there is no clinical data on a hypofractionated treatment of the pelvic lymph nodes. However, dose-escalated treatment of the pelvic nodes with 56 Gy in conventional fractionation combined with hypofractionated treatment of the prostate (70 Gy in 2.5 Gy fractions) has been shown to be well tolerated [25].

We chose a fractionation regimen with 54 Gy in 3 Gy fractions to the prostate bed with or without the treatment of the pelvic lymph nodes with 45 Gy in 2.5 Gy fractions. Assuming an α/β of 1.5 Gy, the BED is 69.4 Gy for the prostate bed and 51.4 Gy for the pelvic lymph nodes. These prescriptions are well below the tolerance doses of the respective organs at risk such as small bowel (BED 47.5 Gy, α/β 7 Gy) and rectum (BED 63 Gy, α/β 4 Gy). If, contrary to recent clinical data, the α/β for prostate cancer is as high as 10 Gy, our prescription would result in a BED of 58.5 Gy to the prostate bed and 46.9 Gy to the lymphatic drainage.

This phase II trial was initiated to substantiate the data on safety and efficacy of postoperative hypofractionated irradiation of the prostate bed, to investigate for the first time hypofractionated treatment of the pelvic lymph nodes and to compare toxicity rates of hypofractionated treatment of the prostate bed alone or with the lymphatic drainage. A safe and effective hypofractionated regimen is attractive for radiation oncology facilities and for patients: While treatment facilities benefit from a higher throughput, patient comfort is increased by a shorter treatment duration.

## Abbreviations

ARO: Arbeitsgemeinschaft Radiologische Onkologie; ASTRO: American Society for Radiation Oncology; BED: Biologically Effective Dose; BfS: Bundesamt für Strahlenschutz; BFS: Biochemical Recurrence-Free Survival; CRF: Case Report Form; DEGRO: German Society of Radiation Oncology (Deutsche Gesellschaft für Radioonkologie); EORTC: European Organisation for Research and Treatment of Cancer; GI: gastrointestinal; GU: genitourinary; HT: Helical Tomotherapy; IMRT: Intensity-Modulated Radiotherapy; ITT: Intent To Treat; MV-CT: Megavoltage-CT; NCI CTC AE: National Cancer Institute Common Terminology Criteria for Adverse Events; PSA: Prostate-Specific Antigen; PTV-P: Planning Target Volume-Prostate Bed; PTV-L: Planning Target Volume-Lymph Nodes; QoL: Quality of Life; RTOG: Radiation Therapy Oncology Group; SWOG: Southwestern Oncology Group; WPRT: Whole-pelvis Radiotherapy.

## Competing interests

The department of Radiation Oncology, University Hospital Heidelberg has a research cooperation with Accuray Inc. (Sunnyvale, CA, USA).

## Authors’ contributions

SK was responsible for drafting the manuscript and trial protocol. FS, KH and LE were involved in critical revision of the manuscript and trial protocol. LE was responsible for the statistical design. DN and FS were responsible for radiotherapy planning and treatment. JD has given final approval of the manuscript. All authors read and approved the final manuscript.

## Pre-publication history

The pre-publication history for this paper can be accessed here:

http://www.biomedcentral.com/1471-2407/12/504/prepub
